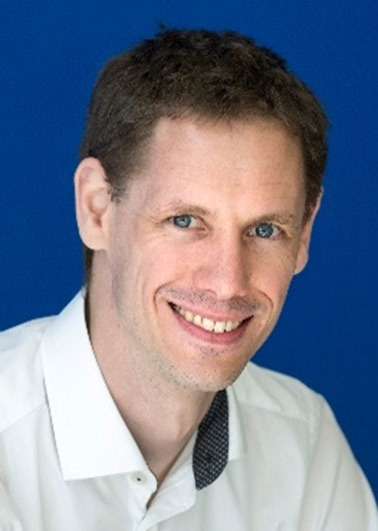# Opportunities for Expanding Encoded Chemical Diversification and Improving Hit Enrichment in mRNA‐Displayed Peptide Libraries

**DOI:** 10.1002/cbic.202100685

**Published:** 2022-02-18

**Authors:** Paddy R. A. Melsen, Ryoji Yoshisada, Seino A. K. Jongkees

**Affiliations:** ^1^ Department of Chemistry and Pharmaceutical Sciences VU Amsterdam De Boelelaan 1108 1081 HZ Amsterdam The Netherlands

**Keywords:** bioorthogonal chemistry, DNA-encoded libraries, drug discovery, mRNA display, peptide drugs

## Abstract

DNA‐encoded small‐molecule libraries and mRNA displayed peptide libraries both use numerically large pools of oligonucleotide‐tagged molecules to identify potential hits for protein targets. They differ dramatically, however, in the ‘drug‐likeness’ of the molecules that each can be used to discover. We give here an overview of the two techniques, comparing some advantages and disadvantages of each, and suggest areas where particularly mRNA display can benefit from adopting advances developed with DNA‐encoded small molecule libraries. We outline cases where chemical modification of the peptide library has already been used in mRNA display, and survey opportunities to expand this using examples from DNA‐encoded small molecule libraries. We also propose potential opportunities for encoding such reactions within the mRNA/cDNA tag of an mRNA‐displayed peptide library to allow a more diversity‐oriented approach to library modification. Finally, we outline alternate approaches for enriching target‐binding hits from a pooled and tagged library, and close by detailing several examples of how an adjusted mRNA‐display based approach could be used to discover new ‘drug‐like’ modified small peptides.

## Graphical abstract

**mRNA‐displayed libraries** of peptides and DNA‐encoded libraries of small molecules are compared and contrasted in this review with a focus on diversification reactions and selection approaches where the former can learn from developments in the latter. We conclude by outlining a path towards approaches that may offer discovery of hybrid small‐molecule peptidomimetics.

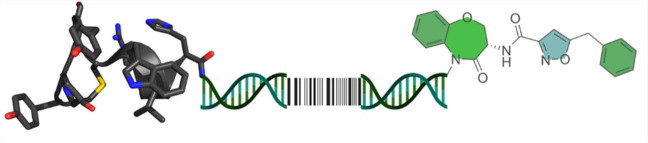

## 1 Introduction

Messenger RNA display (mRNA display) uses a covalent connection between a peptide and its encoding RNA to generate numerically large libraries of peptides. The templated amide‐bond forming ability of the ribosome is harnessed in generating the library, and so allows access to relatively large molecules in a convenient manner but with a limited number of different building blocks, and the attached nucleic acid sequence can be used to reliably decode which sequence(s) bound to a target.^[1]^ DNA‐encoded small‐molecule libraries (DEL) also use a covalent connection between DNA and a molecule of interest to identify hits from a library, but in this case the library is generated on (or concomitantly with) the DNA tag using carefully optimized selective chemistry. This allows a much broader range of building blocks to be used, but can make generation of a new library more time‐consuming (although re‐use of libraries means this is not often needed).^[2]^ These two approaches thus share many aspects and have complementary strengths. In recent years a trend has developed for the use of selective chemical reactions to modify mRNA‐displayed peptides following translation, bringing these two fields closer. Note that, while mRNA displayed peptide libraries could fall under the umbrella of DELs, in this work they will be discussed as distinct entities, with DELs here explicitly being taken to exclude libraries of peptides composed of canonical amino acids.

Peptides as drugs have advantages in high selectivity and efficacy and a relatively non‐toxic nature, but suffer from unfavorable physicochemical properties that give rise to their tendency to aggregate, low oral bioavailability, short half‐life/fast elimination, low membrane permeability, and susceptibility to hydrolysis and/or oxidation. All of these impose significant challenges in a therapeutic environment.^[3]^ By generating a molecule intermediate between a small molecule and a peptide, we may be able to access the best of both worlds – a molecule that has favorable pharmacokinetic properties but still has a sufficiently large binding surface to bind diverse protein types and access to diverse three‐dimensional folds for scaffolding functional groups. While the exact ‘rules’ for what allows a peptide or peptidomimetic to be orally available are less well defined than those for traditional small molecules (e. g. the so‐called ‘rule of 5’^[4]^ and variations thereof), the same parameters of molecular weight, hydrogen bond donors and acceptors, and lipophilicity remain important, with one review reporting an 80^th^ percentile cut‐off of 1216 Da, LogP of 5.1, 23 hydrogen bond acceptors and 9 hydrogen bond donors for oral peptide drugs.^[5]^ By limiting the size of the peptide backbone and increasing building block chemical diversity through encoded post‐translational modifications a library may be created that maintains the advantages but mitigates some of the disadvantages typically seen with peptides, and so may increase the discovery rate of orally available drug candidates.

In this review we compare and contrast developments in both mRNA display and DELs, with a particular focus on innovations in the DEL field that can also be exploited in mRNA display and with an eventual goal of outlining the possibilities for a hybrid approach to access libraries of such ‘best of both worlds’ molecules. We will briefly explain here the basic workflow of both mRNA display and DELs and some variants that exist within each; detail chemical modification approaches currently employed in mRNA display and survey opportunities for expansion of this on reactive handles from both canonical and non‐canonical amino acid functionalities based on DNA‐ and protein‐compatible chemistry; propose some potential approaches to encode additional information in the nucleic acid tag of an mRNA displayed library to encode multiple modifications; and summarize some advanced methods for enriching target‐binding library members before concluding with a vision on how a hybrid mRNA/DEL approach may look in practice.

## 2 Variants of mRNA Display and DNA‐Encoded Libraries

Both DELs and mRNA display have been reported in various forms, with the shared core concept of connecting an encoding piece of oligonucleotide to a large collection of molecules of interest. These variations can have implications for what may be possible in a hybrid system, and so we will begin by outlining the general approach here followed by separate sub‐sections with the main variants within each technique.

Overall libraries in mRNA display are both numerically larger and contain larger molecules than in DELs, reflecting the ease with which the ribosome can be used to carry out a templated oligopeptide synthesis. Being a peptide display approach, it is typically lower in building block diversity than DELs, but the natural amino acids cover a range of properties that are generally well‐suited to binding to a protein target. DELs by contrast have access to a much broader range of building blocks, but are typically limited to two or three rounds of reaction, except for macrocycle libraries which generally require a larger number of reactions.^[6]^ This is largely by design, reflecting a desire to keep the molecules within ‘rule of 5’ chemical space. Furthermore, the majority of commercially available building blocks consists of primary‐ and secondary amines, carboxylic acids and carbonyl compounds, as shown by an extensive price‐focused analysis.^[7]^ Thus, a numerically large library can certainly also be created with this technique if mainly relying on amide bond formations and reductive alkylations/aminations. Moreover, reagents such as sulfonyl chlorides, boronic acids, aryl halides, alkynes and azides have also been used in DEL constructions,^[8]^ but are less accessible as they are one or two orders of magnitude fewer in commercial availability compared to amines, carboxylic acids and carbonyls.^[2]^ DELs selections are typically carried out as two to three parallel rounds of selection, whereas mRNA‐display is typically done over multiple sequential rounds of enrichment. This allows for ‘molecular evolution’ of peptides through mutagenesis and selective pressure, and can be advantageous when mRNA display libraries cannot cover the full possible sequence space, but is typically not needed for DELs because of their lower numerical size. Finally, in mRNA display the information is encoded in mRNA or an mRNA‐cDNA hybrid duplex while DELs have this in either single‐ or double‐stranded DNA (or occasionally PNA^[9]^), with associated differences in stability. This overview and comparison of mRNA display and DELs is summarized in Table 1.

**Table 1:** Overview comparison of mRNA display and DNA‐encoded small‐molecule libraries.

Property	mRNA display	DNA‐encoded small‐molecule libraries (DELs)
Number of library members	∼10^13^	∼10^5^–10^10^
Nature of tag	ssRNA or dsRNA/cDNA	ssDNA or dsDNA
Type of encoding	Templating	Recording, routing, templating (see Figure 2)
Typical number of building blocks per library member	10+	2–4 (more for macrocycles)
Number of different building blocks	20^[a]^	100,000+^[20][b]^
Building block connections	Amide bonds	Amide bonds, C−C bonds, C−N bonds, sulfonamides, heterocycles, carbocycles
Number of rounds in a typical selection	3–10, sequential	2–4, parallel^[c]^
Selection	Pull‐down	Pull‐down predominates (selective PCR, cross‐linking and gel purification possible)

[a] Expandable to some extent with genetic code reprogramming. [b] Based on an estimate of commercially available building blocks. [c] Multiple sequential round DEL selections are possible, but less needed.

### 2.1 Messenger RNA display

In mRNA display, the peptide library is generated using the mRNA tag as a template for translation. Key to this technique is the antibiotic puromycin, which on its own terminates translation non‐specifically.^[10]^ Puromycin is composed of *O*‐methylated tyrosine connected to *N*6‐dimethylated adenosine through an amide linkage, which makes it stable in most biological settings. Attachment of puromycin to the end of mRNA means that it can enter the ribosome A‐site at the end of translation, where the amino acid moiety is attached to the *C*‐terminus of the peptide chain and thus provides the necessary stable covalent connection between peptide and encoding sequence.^[11]^ This process can be made more efficient by using an *in vitro* translation system deficient in release‐factors to increase the residence time of the mRNA and peptide in close proximity in a stalled ribosome.

In a typical mRNA display experiment for peptide discovery, a DNA template encoding a library is first generated by PCR from synthetic primers, subsequently transcribed and connected to an oligonucleotide with 3’ puromycin, and finally used as a template for *in vitro* translation. The resulting peptide library is enriched by incubation with a target of interest, which is immobilized in some way (e. g. column, plate or magnetic beads), and the library members that bind the target are reverse transcribed (either before or after enrichment) and then amplified by PCR. This generates an enriched DNA library that can be used as an input for one or more additional rounds, with a typical selection campaign taking anywhere between 3 and 10 such sequential rounds. Hits can then be identified by sequencing of the cDNA, which is decoded to identify binding peptides. While mRNA display of proteins is possible, the focus in this review will be on libraries of short peptides.

**Figure 1:**
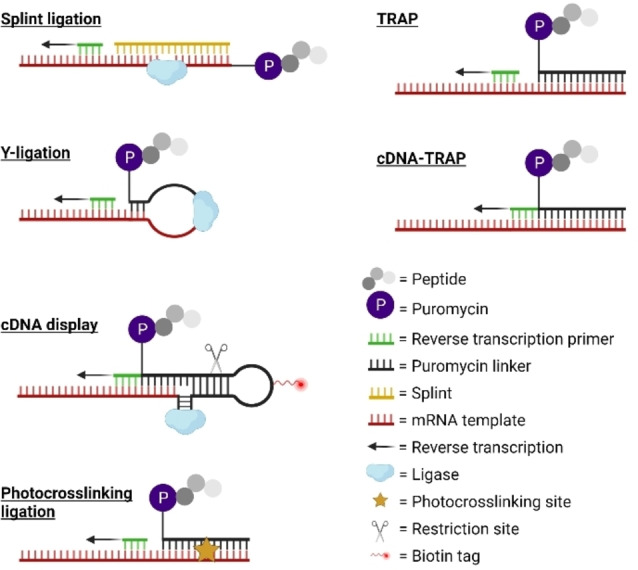
Diverse approaches to puromycin connection and reverse transcription priming in mRNA display. In the splinted ligation, Y‐ligation and photocrosslinking approaches the sequence is encoded in mRNA attached covalently to the peptide; in cDNA display and in cDNA‐TRAP this same information is in DNA attached covalently to the peptide. In the TRAP approach the same information is contained in mRNA but attached non‐covalently through base pairing. Splinted ligation requires additional purification to remove the splint, while cDNA display facilitates library purification through the attached biotin and cleavage site.

The key variation between different approaches to mRNA display is in how puromycin is attached to the mRNA library (Figure 1). Originally, the ligation of puromycin was by an oligonucleotide spacer attached through splinted ligation,^[10]^ but this had low efficiency in peptide capture and required a denaturing gel or nuclease treatment to remove the splint. Substituting a polyethylene glycol spacer for the polyA improved capture efficiency by increased flexibility,^[12]^ and diverse approaches were developed to more conveniently and/or efficiently connect the puromycin to the mRNA. Changing to a Y‐type ligation^[13]^ removes the need for a splint, simplifying the set‐up (termed an ‘in vitro virus’ by the authors).^[14]^ This ligation approach combined with flexible *in vitro* translation allowed incorporation of diverse non‐canonical amino acids (termed the ‘RaPID system’ by the authors).^[15]^ Incorporating a biotin and restriction site in the linker facilitates purification by streptavidin capture followed by cleavage for release. In the same variant, shifting the puromycin from the 3’ end to a branched chain of a modified nucleobase in the linker also allows the same oligonucleotide to be used as the reverse transcription primer and thus encode the peptide information directly in DNA that is covalently attached to the peptide (termed ‘cDNA display’ by the authors).^[16]^ Photo‐crosslinking simplifies purification of the resulting product, but care needs to be taken with the 365 nm light used to activate the psoralen to avoid damage to the nucleic acids (photocrosslinked mRNA display).^[17]^ Finally, annealing alone gives a sufficiently stable connection if the complementary sequence is long enough and this allows a much faster protocol (termed ‘TRAP display’ by the authors).^[18]^ Furthermore, using a branched linker in this context again allows it to serve as reverse transcription primer (termed ‘cDNA‐TRAP display’ by the authors).^[19]^ These various techniques will collectively be referred to as simply ‘mRNA display’ in this review, but in cases where one approach requires special consideration it will be discussed with the name above.

### 2.2 DNA‐encoded small‐molecule libraries

**Figure 2:**
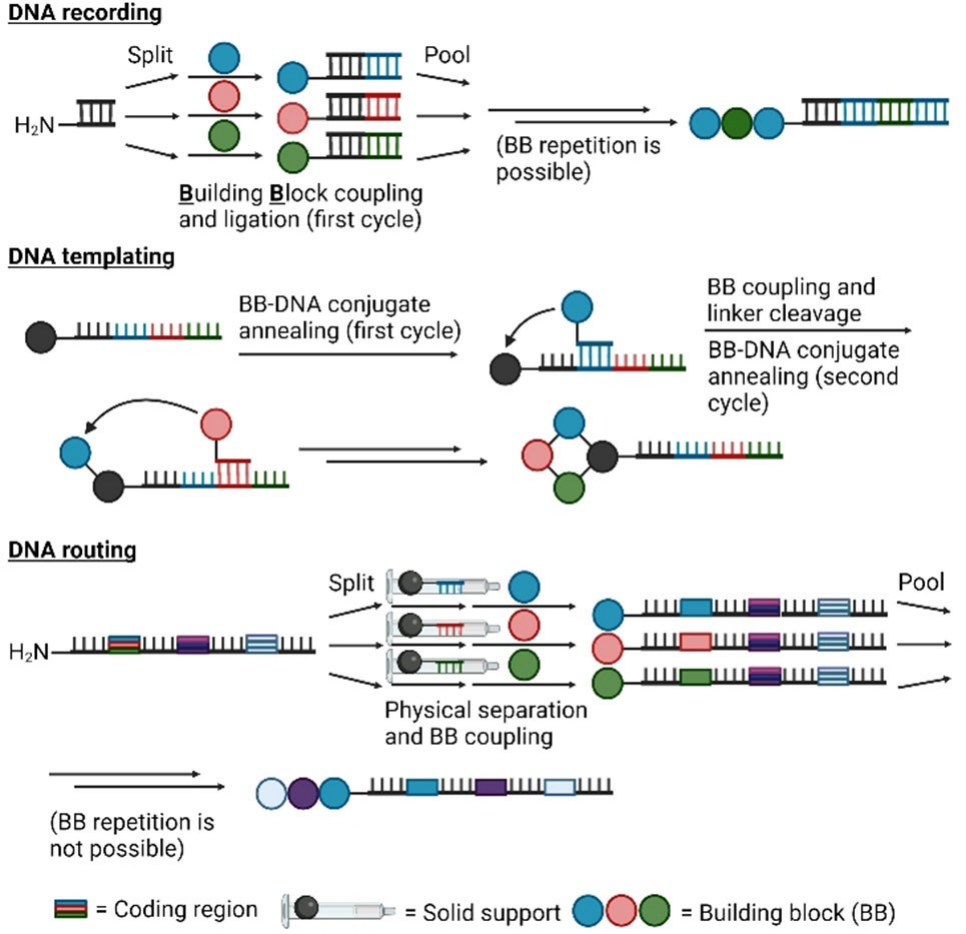
Diverse approaches to DNA‐encoded libraries. In each approach a collection of small molecules is assembled on a DNA tag, but differ in the way in which sequence either records or determines the identity of the molecule. A recorded library typically builds the encoding tag concomitantly with the library member in a split‐and‐pool set‐up; a templated library uses hybridization of oligonucleotide‐coupled building blocks to a template strand to assemble the library member; and a routed approach also uses a split‐and‐pool set‐up but uses hybridization of the library member to an oligonucleotide on a solid support to separate out molecules based on a pre‐existing sequence in the DNA tag before modifying the library member on the solid support.

DNA‐encoded libraries exist in more diverse variants than the sub‐types of mRNA display, both in the chemistry used for library generation and in the way structural information is encoded, and so are harder to describe in a general way. Overall, a small molecule is generated using a small number of reactions with predictable orthogonality (based on experimental validation such as LC‐MS), typically 2–4 reactions but in some cases more and in some cases employing protecting group chemistry, and the sequence of steps for building the library is encoded in an attached single‐ or double stranded DNA molecule. Library generation typically provides ample material for many selections, in which the libraries are enriched for binders to a target of interest followed by sequencing and decoding of the tag (Figure 2). The DNA tag can be generated by ligation of a new fragment during each step of synthesis of the small molecule in a split‐and‐pool approach (DNA‐recorded library^[21]^), can be used as a template to assemble oligonucleotide‐conjugated building blocks (DNA‐templated library^[22]^), or can be used to separate the oligonucleotide pool into distinct reaction vessels based on short complementary sequence tags (DNA‐routed library^[23]^). These are discussed further in section 4.1 below, from the perspective of how such approaches could be integrated with mRNA display. Synthesis can take place in solution or on solid phase, and from solid support can be cleaved for in‐solution enrichment of binders (but not necessarily). In many cases an amine‐modified oligonucleotide is used as a convenient handle to start the synthesis (‘headpiece’), and so amine modification chemistry is a very common reactivity in use for generation of these libraries (but far from the only chemistry). DNA‐recorded small‐molecule libraries are the most commonly used form of DEL, and these typically do not use the output DNA to generate a new library in the same way as in mRNA display (although this is possible by hybridization of the output DNA with the initial library^[24]^). Unlike mRNA display, ‘molecular evolution’ over multiple sequential rounds through mutagenesis and enrichment does not typically take place with a DEL. This is because the number of library members in an mRNA‐displayed library typically does not approach even close to the theoretical diversity, meaning this approach allows sampling of molecules not in the original pool (∼10^13^ library members displayed vs 3.3×10^19^ theoretical 15‐mers). This is not needed with a DEL, where coverage is typically high. Selections with DELs are, however, often multiple parallel rounds to minimize artifacts. In a DEL, the correlation between sequence and building block is largely arbitrary, and so each ‘codon’ can be expanded to encode as much information as needed. Codons are typically designed such that a frameshift cannot change one to another.^[24]^

## 3 Chemistry Compatible with Nucleic Acid Tags

Approaches for selective modification of peptides and proteins are well‐established, but the field also continues to innovate.^[25]^ The modification of a peptide library in an mRNA display format has some specific challenges because of the low concentration of the library (low to sub‐micromolar) and the need to avoid damage to the nucleic acid tag or its connection to the peptide. Nonetheless, several methods have been established as compatible with all of these factors and already implemented in mRNA display (discussed in section 3.1), while further reactions that have been described for selective modification of peptides or proteins suggest themselves as promising areas for expansion (discussed in sections 3.2 and 3.3, covering reactions using canonical or non‐canonical amino acid functionality as a handle, respectively). Some of these further reactions have already been shown to be DNA‐compatible or deployed in DEL construction,^[8]^ and some have much more diverse pools of suitable building blocks available,^[26]^ and these will be emphasized accordingly. It should be noted here that not all DNA‐compatible reactions will be RNA‐compatible, and for this reason the appropriate choice of mRNA‐display approach may be important for some reactions to be successfully employed (for example cDNA display and cDNA‐TRAP, which encode the peptide sequence in a covalently attached DNA molecule). Notably, more aggressive conditions can at times be employed than are compatible with proteins, such as relatively high levels of organic co‐solvents, since peptides are more dynamic than proteins and so relevant conformations are more easily re‐established. Modification reactions described to date in mRNA display have typically been targeted changes to the peptide to bring in a specific functionality, rather than a diversity‐oriented approach, but this aspect will be further discussed below (section 5). We will focus here on approaches that are able to add additional functional groups to a library to increase chemical diversity, and minimize discussion of approaches that are only useful in cyclization^[1]^ or limited in scope^[26]^ (reviewed elsewhere).

### 3.1 Chemistries demonstrated to selectively modify mRNA‐displayed peptides

**Figure 3:**
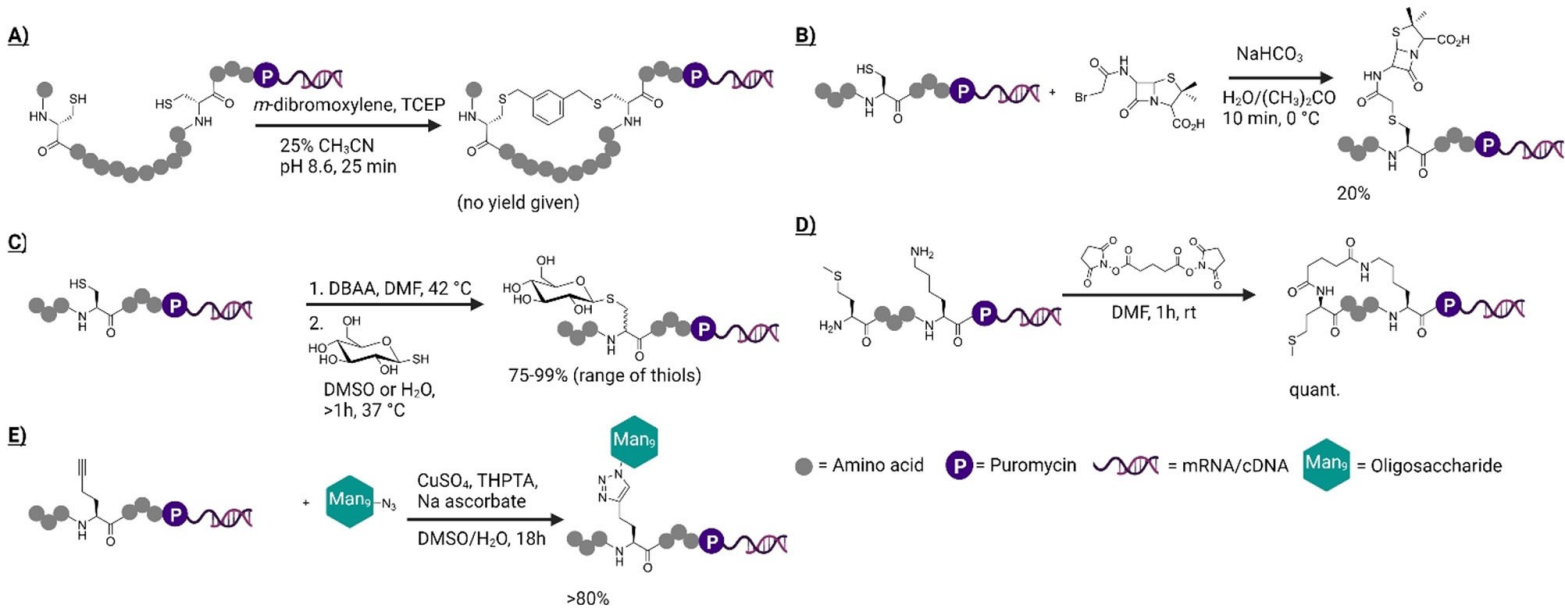
Chemistries demonstrated to selectively modify mRNA‐displayed peptides, by A) cysteine crosslinking with a bifunctional aryl halide, B) cysteine alkylation, C) cysteine conversion to dehydroalanine then conjugate addition, D) amine crosslinking with a bifunctional NHS ester, E) CuAAc reaction on an alkyne amino acid (methionine analog).

Thiol modification is a common approach for chemical modification of peptide libraries in mRNA display, exploiting its high nucleophilicity. Crosslinking of cysteine residues with dibromo‐*m*‐xylene (DBX) was developed by Timmerman *et al*. for peptide modification,^[27]^ and later applied in mRNA display by Szostak and co‐workers (Figure 3A).^[28]^ Given that the first step of this cyclization is an intermolecular reaction, this reaction should be well suited to the broader diversification of a peptide library. Multiple such thiol‐reactive reagents have been shown by Heinis and co‐workers to be compatible with phage display, giving access to varied linkers.^[29]^ α‐Halogenated carbonyl thioalkylation (e. g. iodo‐acetamide derivatives) is another such well‐established reaction for controlled cysteine modification.^[30]^ In particular, peptide translation initiated by an *N*‐chloroacetylated amino acid is often exploited to form a thioether bond with a downstream cysteine residue^[31]^ in the RaPID system, but this reaction has also been used by Li and Roberts to install penicillin on an mRNA‐displayed peptide library by using a bromoacetyl alkylation on a cysteine side chain (Figure 3B).^[32]^ Cysteine can also be used in a bis‐alkylation/elimination reaction to form dehydroalanine.^[33]^ Reagents such as phenylthiosulfonates and dibromoadipic bisamide (DBAA) can be used in peptides and proteins for this conversion,^[34]^ and the resulting dehydroalanine can then undergo a diverse range of reactions notably including conjugate addition with thiols (selenols^[35]^ and amines^[36]^ have also been used in proteins), with a carbohydrate‐peptide conjugation having been shown to be compatible with an mRNA displayed peptide (Figure 3C).^[37]^ Dehydroalanine was also shown to be accessible from selenocysteine using hydrogen peroxide in peptides from *in vitro* translation, forming an approach compatible with other cysteine modifications.^[38]^ Dehydrobutyrine was also shown to be accessible in a translated peptide from vinylglycine, but the thermal isomerization step required may be too harsh for an mRNA tag.^[39]^

An amine is also a common and useful handle for selective chemical modification of a peptide or protein. The most commonly used reagent for this is an *N*‐hydroxysuccinimide (NHS) ester, but several other suitable leaving groups also exist that improve solubility or aqueous stability (e. g. sulfo‐NHS and pentafluorophenol).^[40]^ In an mRNA displayed library, Roberts and co‐workers have used disuccinimidyl glutarate to cyclize a peptide by bridging two amines (lysine or *N*‐terminus, Figure 3D).^[41]^ As with the thiol‐bridging cyclization above, the first step of this is an intermolecular reaction. Notably, many such activated esters can be purchased or easily synthesized, and they leave only a small amide linker in the final product. This does, however, introduce hydrogen bond donors and acceptors. NHS esters can occasionally also react with hydroxyl groups with the assistance of a surrounding catalytic environment.^[42]^ However, the use of this catalytic effect in a peptide context is limited since peptide folding in the aqueous phase is extremely difficult to control in library design, and so likely represents a possible side‐reaction more than an exploitable effect.

Finally, the copper‐catalyzed azide‐alkyne cycloaddition (CuAAc), also known as a ‘click’ reaction, has also been exploited for library modification in mRNA display. To allow CuAAc, Krauss and co‐workers used a non‐standard amino acid with an alkyne on its side chain (homopropargylglycine) to replace methionine in a library of peptides, and this was used for addition of multiple instantiations of a complex glycan per sequence (Figure 3E).^[43]^ This reaction has also been shown to be suitable for cyclization after *in vitro* translation,^[44]^ and because the CuAAc reaction is orthogonal to many other reactions it can also be used for bicyclic peptide libraries. Notably, Hartman and co‐workers have shown that both CuAAc and thiol cross‐linking with dibromoxylene can be used together in mRNA display.^[45]^ This then sets the scene for using multiple modification reactions on a peptide to introduce additional diversity.

### 3.2 Other chemistries not yet demonstrated in mRNA display, using canonical amino acids as a handle

**Figure 4:**
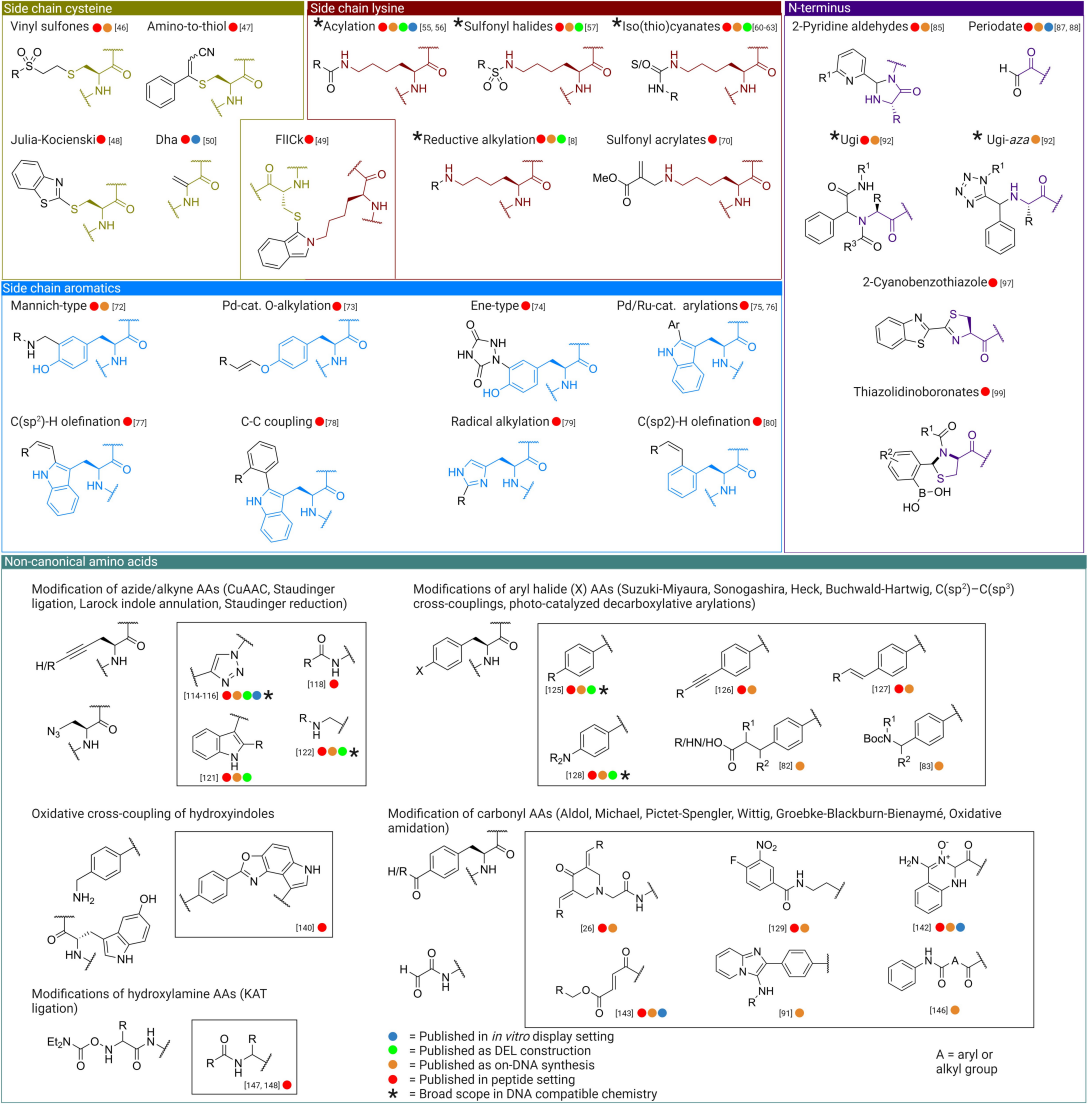
Modification reactions from outside mRNA display literature that are likely compatible with peptide library diversification in mRNA display, using functional groups from canonical (cysteine, lysine, aromatic amino acids and the *N*‐terminus – each highlighted in the respective structures) and non‐canonical (structures as shown) amino acids. Type of source literature is indicated by colored circle, and reactions that have a particularly broad scope in DNA‐compatible chemistry are emphasized with a star.

While the reactions described in the previous section demonstrate the principle of chemical diversification of peptide libraries, there is a much broader pool of selective chemistry that may also be exploited for this goal but which has not yet been tested in this setting. Here we draw on reactions shown to be compatible with proteins and peptides, but not in all cases with an RNA or DNA tag (summarized in Figure 4).

Of the canonical amino acids, cysteine is typically the most convenient handle for selective functionalization of peptides and proteins (and has already been exploited in mRNA display), with a very common reagent being maleimide derivatives. However, in the context of a drug discovery setting for small molecule‐peptide hybrids this reaction is much less appealing as the resulting thioether bond is susceptible to oxidation, the connection is reversible, multiple hydrogen‐bond donors are introduced and the maleimide is a relatively bulky group that is unlikely to contribute to target binding. Examples of more suitable reaction partners for diversity‐oriented modification of peptides to give stable modifications include vinyl sulfones for conjugate addition (although still reversible, less bulky);^[46]^ 3‐arylpropionitriles for amine‐to‐thiol conjugation reactions;^[47]^ a methylsulfonyl benzothiazole for modified Julia‐Kocienski olefination;^[48]^ and a fluorescent iso‐indole crosslinking reaction.^[49]^ A particularly promising handle for diversification is conversion of cysteine to dehydroalanine, which results in a uniquely electrophilic moiety in a peptide context with minimal spacer.^[50]^ As mentioned in section 3.1, this can undergo conjugate addition reactions, but also Diels‐Alder reactions^[51]^ and act as alkene substrate for transition‐metal catalyzed cross‐coupling reactions or radical additions^[52]^ (although with the associated danger of DNA damage).^[53]^ Diels‐Alder reactions are especially suitable for diversification as they have been validated in library synthesis and generated hit compounds for tumor necrosis factor (TNF)^[54]^ and carbonic anhydrase‐IX (CA‐IX).^[55]^ However, worth noting is the fact that both libraries were based on a more reactive maleimide derivative for the dienophile.

Another convenient and commonly exploited functional group for selective modification in peptides and proteins is the primary amine in the side‐chain of lysine, which can be reacted selectively over that of the N‐terminus by appropriate pH control and over the cysteine thiol by use of harder electrophiles. Easily the most accessible modification in DELs is an amide bond formation, especially considering amines and carboxylic acids make up the majority of relevant commercially available building blocks. Hence it is no surprise that both compounds currently in late‐stage clinical trials that have been obtained from DELs, a Receptor Interacting Protein 1 Kinase^[56]^ and a soluble epoxide hydrolase (sEH) ligand,^[57]^ contain amide bonds obtained from acylation. Aside from acylations and the NHS esters mentioned in the previous section, examples of reactions on amines include sulfonyl chlorides;^[58]^ ketenes,^[59]^ isothiocyanates^[60]^ (or in situ‐generation of isothiocyanates)^[61]^ and isocyanates, the last having been used in the construction of libraries against ADAMTS‐5 metalloprotease,^[58]^ thrombin^[62]^ and mitochondrial branched chain aminotransferase (BCATm).^[63]^ These reactions all are used under neutral or mildly basic conditions and moderate temperatures. Furthermore, reductive alkylations also offer specificity towards lysines, although these were historically slow in the aqueous conditions which are typically required in an mRNA‐display format. Numerous improvements have been made to protocols that now allows for easier incorporation of this reaction in DEL construction.^[8]^ As a result, numerous libraries have been synthesized using reductive alkylation/amination for the discovery of novel hits against phosphoinositide 3‐kinase α (PI3Kα),^[64]^ sEH,^[65]^ *N*‐α‐Acetyltransferase 50,^[66]^ and BCATm.^[63]^ One approach for constructing branched products in DELs is by using the triazine scaffold provided by cyanuric chloride. Many libraries have been constructed this way and enriched against numerous targets such as ADAMTS‐4,^[67]^ ADAMTS‐5,^[58]^ she,^[57]^ Lymphocyte function‐associated antigen 1^[68]^ and Oxacillinase‐48 Carbapenemase.^[69]^ However, in the context of this review, we see the triazine scaffold as inspiration but likely not directly relevant since the peptide would serve the same purpose. More recently a method has been developed to distinguish between different lysine side‐chains in a protein context, with a sulfonyl acrylate that reacts chemo‐ and regioselectively with the computationally‐predicted most reactive lysine (lowest p*K*_a_).^[70]^ Furthermore, the resulting derivatives provide a new acrylate handle for further modification through for example conjugate additions or Diels‐Alder reactions. Also worthy of mention is the use of imidazole‐stabilized sulfones for the preparation of azides,^[71]^ although this is only a functional group interconversion and does not directly build diversity.

A growing body of selective reactions have been described that modify the sidechains of aromatic amino acids, especially the electron rich rings of tyrosine and tryptophan in C(sp^2^)−H functionalizations and transition‐metal catalysis, while tyrosine also offers some additional possibilities to exploit the nucleophilic hydroxyl (again with pH control for selectivity). Methods for the modification of tyrosines include Mannich‐type reactions,^[72]^ Pd‐alkylation,^[73]^ and ene‐type reactions.^[74]^ Tryptophan can undergo a series of C−H activations such as Ru‐^[75]^ and Pd‐catalyzed^[76]^ *C*2‐arylations; *C*2 olefination;^[77]^ and C−C coupling with aryl halides.^[78]^ Histidine^[79]^ and phenylalanine^[80]^ have also received increasing attention as handles for selective modifications, but these modifications are fairly new in the field of peptide chemistry, where they are carried out in organic media and so likely are not yet compatible with mRNA or DNA. Nevertheless, there have been a number of C(sp^2^)−H^[81]^ and C(sp^3^)−H^[82]^ activations using Pd/Ru‐catalysis and C−H alkylations promoted by visible light^[83]^ on other residues in a DNA‐compatible format, which leads us to believe that in the future these reactions may well be adapted into an aqueous and DNA/mRNA‐compatible format.

The *N*‐terminus is unique in its reactivity and can hence be utilized as a handle for a number of chemical post‐translational modifications on top of the amine modifications used with lysine,^[84]^ although it is not always accessible for chemical modification (for example remaining formylated in some bacterial‐derived *in vitro* translation systems). One such unique functionality of the *N*‐terminus is the mild nucleophilicity of the downstream amide bond to trap transient adducts. This can be efficiently exploited in protein modification using 2‐pyridine aldehydes to form imidazolidinones,^[85]^ and this should be equally applicable in mRNA display. This reaction is appealing since it is independent of the nature of the amino acid, with the only exception being proline in the second position, and generates a heterocyclic product with fewer hydrogen bond donors than the original peptide. An enzymatic strategy could also be employed at this reaction site, using a ligase to bring in a short peptide fragment with new functional groups or chemical handles. A ligase with broad specificity such as the engineered subtilisin derivative omniligase‐1 would be particularly suited.^[86]^

A commonly deployed strategy with *N*‐terminal serines and threonines is oxidation with periodate, which yields a reactive aldehyde. Such an aldehyde can in turn be decorated with new moieties by reductive alkylations and oxime‐^[87]^ or hydrazine‐ligations.^[88]^ Other examples of aldehyde‐based diversification from DNA‐compatible chemistry that offer significant promise from a drug discovery perspective include a report on construction of a large variety of heterocycles,^[60]^ including newly developed protocols for the preparation of isoquinolones,^[89]^ oxindoles^[90]^ and thiazole‐fused dihydropyrans;^[91]^ and a number of isocyanide‐based multicomponent reactions.^[92]^ Note that a solid‐support strategy is used for these reactions to enhance DNA‐stability, which has also been deployed in mRNA display‐based methods (mentioned in section 4.1 below). Indeed such a solid‐support strategy has been more broadly exploited to expand the scope of DNA‐compatible chemistry and is not limited to on‐resin synthesis – DNA can be captured reversibly from solution onto quaternary ammonium support to carry out reactions in organic solvents.^[93]^ Using a solid support approach it was shown that aldehydes in particular can undergo an ‘explosion’ of diversity from one precursor into many different structures (including olefins, alkynes, amides, amines, β‐hydroxy‐ and β‐amino‐ketones, and diverse heterocycles).^[94]^

*N*‐terminal cysteines also offer possibilities for further modification,^[95]^ with native chemical ligation being an obvious example that can be used to attach diverse thioester precursors to the *N*‐terminus.^[96]^ Nitrile‐based reagents also provide an option for clean *N*‐terminal modification in proteins and should be compatible with mRNA display, for example cyanobenzothiazoles^[97]^ (although some sequence‐specific reactions with other cysteines are possible and cannot be neglected in a peptide library setting).^[98]^ Gao *et al*. designed a particularly promising approach for the incorporation of thiazolidinoboronates in peptides that provides new diversity at several positions as well as generating an additional reactive handle in the form of a boronate.^[99]^ Considering the increasing use of transition‐metal catalysis in DNA‐encoded chemistry, this boronate handle could prove to be an interesting option for building diversity through sequential reactions such as Suzuki‐Miyaura^[100]^ and Heck cross‐couplings.^[101]^ Additionally, it was observed that the previously discussed bis‐alkylation/elimination reagent DBAA (used for the preparation of dehydroalanines from cysteines) is also capable of generating a ketone from N‐terminal cysteines,^[37]^ which can be used as an orthogonal handle for further diversification.

### 3.3 Other chemistries not yet demonstrated in mRNA display, using canonical amino acids as a handle

Because mRNA display generates a peptide library using an *in vitro* translation system it is particularly amenable to genetic code reprogramming. Various approaches for charging non‐canonical amino acids can be used in this setting, including the use of near‐cognates of canonical amino acids;^[102]^ evolved aminoacyl‐tRNA synthetase and tRNA pairs;^[103]^ ligation of chemically acylated pdCpA dinucleotides onto truncated tRNA;^[104]^ and acylation of *in vitro* transcribed tRNA with chemically activated amino acids through the use of acylating ribozymes called flexizymes.^[105]^ Creation of a vacant codon is similarly convenient in this setting, including by sense codon reprogramming through omission of canonical amino acids and/or amino‐acyl tRNA synthetase enzymes;^[106]^ stop codon suppression (enhanced by the omission of release factors that also improves puromycin capture);^[107]^ antisense suppression of specific tRNA of canonical amino acids;^[108]^ and suppression of formylation to liberate the initiation codon.^[109]^ Much work has been done to use genetic code reprogramming in the context of mRNA display to directly influence the drug‐like parameters of the peptides, for example by translation of d‐amino acids,^[110]^ methylated amino acids,^[111]^ and peptoid building blocks.^[112]^ While we will discuss reactions that use a functional group handle introduced on a non‐canonical amino acid through genetic code reprogramming, we will not discuss increasing peptide diversity directly through the use of that technique, although such approaches certainly can achieve some of the same goals and likely would be compatible with extensive covalent modification (*N*‐methylation and peptoid building blocks in particular likely having synergy in shifting the chemical space towards more drug‐like molecules).

Copper‐catalyzed azide‐alkyne cycloaddition (CuAAc) is a very commonly used selective reaction that is compatible with both proteins and nucleic acids, although some risks exist for side‐reactions of oxidized ascorbic acid with lysine and arginine sidechains and reactive oxygen species giving damage in DNA (both can be mitigated with appropriate scavengers).^[113]^ Notably, this has already been employed in mRNA display (see section 3.1 above). Several small molecule libraries using CuAAc chemistry have been published, including a library based on a benzodiazepine and a pyrazolopyrimidine scaffold^[114]^ and two libraries consisting of macrocyclic compounds, which were screened against Human Serum Albumin (HSA) and α‐1‐acid glycoprotein (AGP)^[115]^ in one case and CA‐IX, Horseradish peroxidase, tankyrase 1, HSA, AGP, Calmodulin, human prostate‐specific antigen and TNFα in another.^[116]^ Both the azide and alkyne functional groups can be conveniently accessed as methionine analogues in azidohomoalanine and homopropargylglycine, respectively, but aromatic azides and alkynes can also be translated to give more reactive versions.^[117]^ A diverse array of azides and alkynes are conveniently available for broad diversification. These reactive handles can also be used in other chemoselective reactions such as (traceless) Staudinger‐Bertozzi ligations,^[118]^ thioacid‐azide amidation,^[119]^ copper‐free click reactions^[120]^ (although perhaps not particularly suited to generation of a drug‐like library due to the high molecular weight of the reagents), Larock indole annulations (which was used for the discovery of a new class of indoleamine 2,3‐dioxygenase‐1 inhibitors using DEL screening),^[121]^ or serving as masked amines following Staudinger reduction^[122]^ (for example to allow for selectivity in sequential amide couplings). Similar to alkynes, alkenes also offer the possibility for selective modification reactions in Inverse‐Electron‐Demand Diels‐Alder reactions^[123]^ and olefin metathesis,^[124]^ although the latter for now suffers from poor conversion in on‐DNA syntheses and so it may be premature to attempt to apply it in mRNA display.

Halogen‐modified amino acids such as iodobenzenes are especially useful for the inclusion of metal‐catalysis in peptide‐chemistry, such as Suzuki‐Miyaura,^[125]^ Sonogashira,^[126]^ Heck,^[127]^ and Buchwald‐Hartwig cross‐couplings,^[128]^ as well as C(sp^2^)−C(sp^3^) cross‐couplings of aryl halides with cyclopropanoic acid and cyclobutylethanone derivatives through Pd‐catalyzed C(sp^3^)−H activation^[82]^ and photo‐catalyzed decarboxylative arylations,^[83]^ which all have been successfully deployed in on‐DNA synthesis.^[129]^ Especially Suzuki‐Miyaura and Buchwald‐Hartwig (and Ullmann) cross‐couplings have been optimized to better fit a DNA‐compatible format. For instance, Suzuki‐Miyaura couplings were limited to on‐DNA aryl iodides when published in that context in 2015.^[130]^ Consecutive improvements to the catalytic system allowed for the inclusion of pyrimidine‐based scaffolds and otherwise unreactive aryl chlorides,^[131]^ as well as easier handling overall.^[100]^ To this day the Suzuki‐Miyaura cross‐coupling has been deployed four times in DEL construction, against BCATm,^[63]^ Polo‐like kinase 1 (PLK1),^[132]^ sEH^[65]^ and PI3Kα.^[64]^ DNA‐compatible protocols for Buchwald‐Hartwig cross‐couplings have also been published,^[133]^ where aryl iodides were coupled to aromatic primary amines using Pd‐catalysis and both amino acids and aliphatic primary amines using Cu‐catalysis. This method was fairly restricted in scope as there were no heteroaryls nor different halides used, and amines were limited to primary amines. Furthermore, DNA degradation was observed in multiple instances. A milder approach was developed compatible with aryl bromides and aromatic amines using precatalyst *t*‐Butyl‐XPhos‐G3.^[134]^ Nevertheless, this method also had its limitations. For example, the aromatic amines could not bear hydroxyls, thiols, or carboxylic acids due to the strongly basic conditions. Furthermore, only nonsterically hindered aryl bromides and anilines gave products with acceptable yields. Despite such limitations, the option to choose freely between aryl bromides and aryl iodides increases options for DEL diversification. The most recent advancement describes a new catalytic system utilizing a PEPPSI catalyst (pyridine‐enhanced precatalyst preparation stabilization and initiation),^[135]^ which have allowed the scope of (hetero)aryl halides to extend to (hetero)aryl chlorides. Furthermore, simple aromatic amines such as aniline; challenging aromatic amines such as *N*‐methyl aniline; and cyclic secondary aliphatic amines such as 3‐phenylpiperidine all provided acceptable yields. Regrettably, the system was unable to provide proper conversion of acyclic secondary aliphatic amines or indoles, which is attributed to β‐hydride transfer side reactions.^[135]^ Libraries generated using Buchwald‐Hartwig cross‐couplings have been used for selection against PLK1,^[132]^ with preparation of another library reported without any apparent selection yet undertaken.^[135]^ Finally for transition‐metal catalyzed reactions, benzoic acid derivatives can also undergo C−H activation on the aromatic ring for reaction with acrylamides^[81]^ and acrylates.^[136]^ Together these reactions represent a particularly promising pool of untapped resources for peptide diversification towards a more druglike library of molecules by diversity‐oriented synthesis, forming products with no hydrogen bond donors or acceptors and with stable C−C bonds, and have the added benefit of many suitable reagents being commercially available.

Micelle‐mediated chemistry has seen increased application for expanding DNA‐compatible chemistry (and thus is perhaps translatable to mRNA‐display) as it is able to chemically compartmentalize reaction media in a single vessel allowing for more stringent conditions. Hydrophobic parts of the library member are located inside the micelle and the hydrophilic DNA‐tag is located in the aqueous phase. This means that oxidative^[137]^ and harshly acidic conditions^[138]^ can be deployed without damaging the nucleic acid tag. Additionally, more efficient Pd‐catalyzed cross‐couplings have also been described using this approach.^[139]^

An orthogonal fluorogenic oxidative coupling reaction has been reported on peptides from *in vitro* translation under a reprogrammed genetic code (although not yet validated in mRNA display), which takes place between benzylamine and 5‐hydroxyindole (e. g. hydroxytryptophan) by treatment with K_3_Fe(CN)_6_. An advantage of this reaction is that the product has a characteristic fluorescence (354 ex., 460 em.) and it was shown to be useful in both intra‐ and inter‐molecular format and so likely would be well suited to the diversity‐oriented application under discussion here.^[140]^

Carbonyl compounds represent another functionality that has been extensively studied in diversity‐oriented synthesis. While *N*‐terminal aldehydes can be accessed without genetic code reprogramming (detailed in section 3.2), a broader range of aldehydes and ketones (and thus reactivity and selectivity) can be accessed through non‐canonical amino acids (e. g. 4‐acetylphenylalanine and 4‐benzoylphenylalanine).^[141]^ Methods for the modification of these carbonyl compounds by Pictet‐Spengler reaction^[142]^ and a Wittig‐reaction,^[143]^ for example, have each been reported in both phage‐display and also for on‐DNA synthesis of tryptolines^[144]^ and α,β‐unsaturated carbonyl compounds respectively.^[145]^ Furthermore, the Groebke‐Blackburn‐Bienaymé isocyanide multicomponent reaction has also been demonstrated to be compatible with DNA (albeit on a solid‐support).^[92]^ A further example of an aldehyde modification in a DNA‐compatible context is copper‐catalyzed oxidative amidation,^[146]^ which allows modification with poor nucleophiles.

Finally, amide‐bond forming ligations can also be carried out with non‐canonical amino acids. These would have a similar goal as native chemical ligation or enzymatic ligation, but with expanded scope and improved control. The keto‐acid hydroxy acid is one such reaction, but suffers from slow kinetics that may make it less efficient in library generation.^[147]^ Potassium acyltrifluoroborates improve on this aspect, but are synthetically more difficult to access (although this has improved recently).^[148]^ Nevertheless, both seem well suited to genetic incorporation as a chemical handle in a peptide library for diversification.

## 4 Encoding of Additional Parameters in mRNA‐Display Constructs

As illustrated by the broad range of chemical transformations discussed in the previous section, generating diversity is perhaps not the biggest challenge in realizing a hybrid approach between mRNA display and DELs, but rather generating a relevant pool of diverse drug‐like modified peptide candidates and identifying the active hit(s) from such a peptide pool. For this goal, additional parameters need to be able to be encoded in, and decoded from, the nucleic acid tag. In this section we detail several strategies by which this could be achieved.

The following discussion in this section is in large part speculative, but it should be noted that some progress in this idea has already been made by Derda and co‐workers in the field of phage display, with silent encoding of multiple carbohydrate modifications^[149]^ or multiple ‘pharmacophores’ in the peptide coding region (equivalent to a DNA‐recorded library).^[150]^ In that work, multiple libraries were generated, each with a different selective modification of the phage‐displayed peptide library, and the identity of the modification was ‘recorded’ in the DNA sequence encoding a flexible linker at the *C*‐terminus of the peptide. This linker was identical in amino acid sequence across libraries, but because of degeneracy in the genetic code sequencing of the DNA could nonetheless be used to identify which modification was present in each hit. It should be noted that this phage display approach only allowed a single round of enrichment (although with multiple parallel selections), but with careful experimental design and use of high‐throughput sequencing this can be enough to identify hits. We argue here that this concept can be extended in multiple ways in the coding and especially the non‐coding regions of an mRNA display construct to access not only the equivalent of DNA‐recorded libraries but also perhaps of DNA‐routed or even DNA‐templated modifications.

### 4.1 Approaches for encoding additional parameters potentially compatible with mRNA display

The DEL‐derived method most directly applicable to mRNA display is likely a recording approach, in which an encoding section of DNA is attached onto the nascent library member through enzymatic ligation.^[151]^ In mRNA display, a cDNA strand is often generated before enrichment, and this hybrid duplex is amenable to extension in such a manner. Using a ligation approach in this context, a numerically large and structurally diverse library can be obtained through iterative split‐and‐pool procedures where each chemical modification is recorded, with library diversity able to arise in both the peptide sequence (templated in the mRNA) and the small molecule fragments (recorded in the appended nucleic acid fragments). Because of the additional molecular weight added in the chemical steps, it seems prudent for such an approach to start with a shorter peptide sequence than is common in mRNA display of an unmodified peptide. What is then lost in sequence diversity in the peptide can be regained in the overall chemical diversity of the library, meaning total library numerical size can remain approximately the same, and so still leverage the large number of molecules assessed in one display experiment. While ligation of a dsDNA fragment with a ‘sticky end’ is typically most convenient, ssDNA is also possible by splinted ligation.^[116]^ Notably, the generation of a ssDNA tag by this approach may offer additional benefits in the library enrichment step (see section 5).

In a templated approach, a sequence of single stranded DNA (or mRNA) brings an oligonucleotide‐linked building block into close proximity with the reacting partner. In essence this is a non‐ribosomal process analogous to translation. This high effective molarity provides the ability to perform the chemical modifications in a one‐pot format, since complementarity of the template strand and the oligo‐linked building block dictates reactivity.^[22]^ Moreover, with this approach templates can be recombined after each round of selection to chemically evolve the library.^[152]^ A templated approach would likely also make any eventual selection process simpler to carry out, and so more likely to succeed. However, oligonucleotide‐linked building blocks would need to be prepared for each modifying reagents and this initial set‐up would be time‐consuming and may limit the compatible chemistry and throughput. A fully autonomous templated system was developed by the group of David Liu in which they were able to perform a series of acylations on‐DNA without any manual input besides initiation of the system. The concept heavily resembles ribosomal peptide synthesis where the DNA‐template acts as the mRNA, and DNA‐tagged substrates resemble tRNA. A ‘walker’ (a DNA strand complementary to part of the template and the DNA‐tagged substrate) is manually added which initiates the assembly and gradually ‘walks’ along the template to assemble the desired product.^[153]^ The diversity of this system is still limited, but it nevertheless offers a promising outlook into fully automated library syntheses.

A DNA‐routed library^[23]^ we believe captures many of the advantages of a templated approach, but mitigates the disadvantages. In this approach templates are passed through one or more DEAE columns, which are each equipped with a unique ‘anticodon’ that is complementary to one of the mutually exclusive sequences found in an encoding fragment of the template strand, allowing for hybridization. This procedure physically separates the template strands into different compartments depending on sequence identity, which is functionally analogous to the split‐and‐pool/recording approach, but without having to ligate the encoding sequences onto the parent strand to record the reaction carried out. Upon immobilization of the template strand, the first set of building blocks are installed and the mixtures are then pooled for the cycle to be repeated for the remainder of the ‘codons’. A DNA‐routed approach thus allows molecular evolution over multiple rounds of selection if desired, as the template determines the nature of the final displayed molecule; it does not require preparation of oligo‐linked building blocks; and it is also more compatible with higher levels of organic solvents and facilitates purification similar to other solid phase synthesis approaches. Notably, precedent exists for such an approach in the use of polyT resin to capture polyA tagged mRNA‐displayed peptide libraries for efficient cyclization by NHS chemistry on solid support.^[154]^ One limitation of the routed approach, however, is that building blocks and tags cannot be repeated, as there is no temporal control like in a split and pool recording approach.

### 4.2 Potential loci for encoding additional parameters in mRNA display

**Figure 5:**
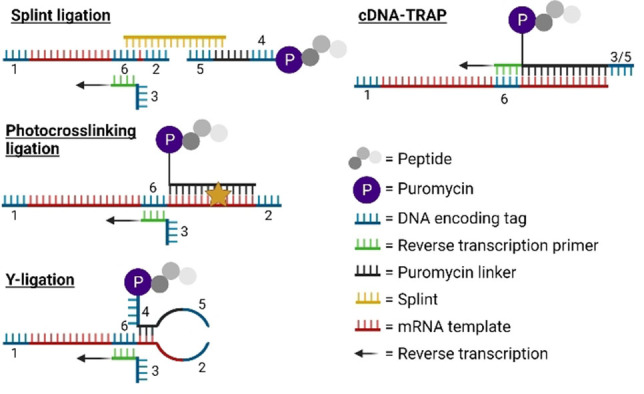
Loci where additional parameters could be encoded in mRNA display (numbered), and how these align with some of the variants for puromycin attachment (see text for a precise definition of each).

The diverse possible approaches to encoding modification reactions in an mRNA‐displayed peptide library described in the previous section all require more parameters to be encoded in the nucleic acid tag. While the encoding of a peptide sequence in the mRNA remains the core of mRNA display, there are multiple possible loci in which more information can be encoded. We outline here what we consider six possible loci for this extra information, and assess how these are compatible with the various encoding approaches above as well as different approaches for mRNA display (Figure 5).

In site 1, information is encoded in a 5’ extension of the mRNA. This would typically be single stranded, but would become a double stranded hybrid duplex after reverse transcription. As such, it is suited to a routing approach, or recording with splinted ligations (as is used at the 3’ end in one approach for attachment of the puromycin linker oligonucleotide). In a double stranded format it would not be possible to achieve an overhang, and so sticky ended ligations would not be possible, although a minority of restriction enzymes are able to cleave an RNA/DNA hybrid duplex and so could find use here.^[155]^ For a templated approach the 5’ end of the mRNA is the most distant part of the oligonucleotide from the peptide, and so this position seems the least suitable of those discussed here to be able to give the needed effective high molarity for this approach.

Site 2 is the 3’ end of the mRNA. In many ways this site behaves the same as site 1, but with control of the reverse transcription primer annealing site this can be directly used for sticky ended ligations with a photocrosslinked or cDNA‐TRAP library. Site 2 likely is also unsuitable for a templated approach because the long puromycin linker, needed to reach the ribosome's peptidyl transfer center, means any building block placed here is still relatively distant from the peptide.^[12]^ Compatibility of this site with a splinted and especially Y‐ligation is likely limited to a few bases, perhaps encoding one reaction, as the efficiency of ligation would otherwise be perturbed.

Site 3 is a 5’ extension of the DNA oligonucleotide used to prime reverse transcription. This would typically be single stranded, but could be double stranded if an additional partially complementary oligonucleotide was added. This would thus be compatible with splinted and sticky end ligations for a recorded library as well as a routed approach, but again seems unlikely to be suitable for a templated library. This site is particularly convenient as it would be simple to prepare a large pool of such oligonucleotides of essentially any length required, and the information is encoded on DNA and so more stable. This site is also broadly applicable to diverse mRNA‐display types, although it is perhaps less suitable for cDNA display and cDNA‐TRAP because of the more involved preparation of the combined puromycin linker/reverse transcription primer. Because of its suitability for splinted and sticky end ligations this site is particularly well suited to repeated rounds of reaction and ligation with a large number of building block in a recorded format.

Site 4 is the encoding of information close to the 3’ end of the puromycin linker (close to the puromycin itself, and thus the peptide). This would necessarily be single stranded, and would be difficult to access for a sequencing read unless the entire linker was an oligonucleotide (which is less efficient in peptide capture by puromycin) and connected to the remainder of the coding region, so likely only suited to splinted or Y‐ligation approaches. Furthermore, synthesis of a diverse pool of puromycin‐containing oligonucleotides may become cost‐prohibitive. While this site clearly has several downsides, it would likely be the most effective site for a templated approach due to its close proximity to the peptide.

Site 5 is an extension to the 5’ end of the puromycin linker. Again, to be able to sequence the information encoded in this site it needs to be connected to the peptide‐encoding region, so it is likely only applicable to a splinted or Y‐type ligation approach to mRNA display (in cDNA‐TRAP this would be the same as site 3). This region was used in cDNA display to include biotin for library purification and release by nuclease cleavage, and so its utility for adding further functionality to an mRNA displayed library has already been demonstrated. This region could contain single or double stranded DNA or RNA (although for a Y‐type ligation requires a few bases unpaired), and so could be applied to any of the above approaches. However, as for site 4 it seems less practical than many of the other sites and without the advantage of high proximity to the peptide.

Finally, site 6 is an extension of the idea behind the silent encoding used by Derda *et al*., with additional information being recorded in the redundant codons typically used to generate a peptide library.^[149]^ For example, the use of NNK, NNS, NNM and NNW codon sets would encode similarly broad peptide sequence space, but would be identifiable after sequencing.^[156]^ Although there is a limitation from redundant codon sets having some overlap (such as ATG falling within both NNK and NNS), in practice it is unlikely with a long enough sequence that only ambiguous codons would be present in a real hit. While possible, this approach would only be useful for low diversity modifications in a recorded approach and does not have any apparent advantages over the others discussed. The previously described silent encoding of reactions in a conserved linker region could, however, be extended to a routed approach, and a larger number of codon combinations are accessible while still encoding the same peptide linker sequence.

In addition to their independent use, several of these approaches could see use in a combined approach. For example, a small number of reactions could be encoded in site 1 (mRNA 5’ end extension) for a set of uniquely tagged libraries that are pooled after initial separate translation and modification and this could be further diversified by a larger number of reactions each encoded by site 3 (a unique reverse transcription primer per reaction vessel), giving easy access to two recorded reactions.

## 5 Selection Methods

A number of alternate hit enrichment strategies have been published for DELs (or model libraries), which we see as also having promise in mRNA display. These will be surveyed in the following section, but it should be emphasized that these have not been extensively applied.

**Figure 6:**
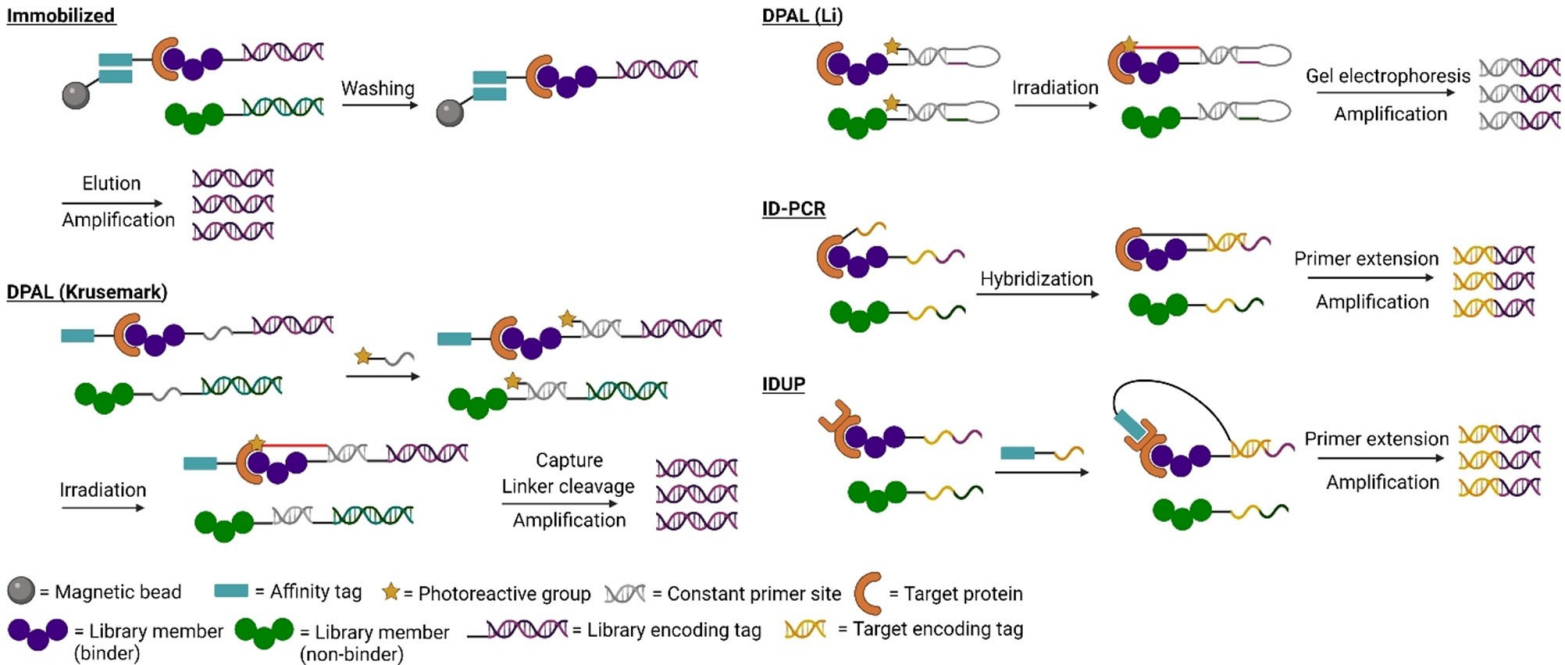
Alternate approaches to hit enrichment from the DEL literature. Immobilized targets are the current standard for both mRNA display and DELs, pulling down interacting hits, but suffer from possible problems such as the need for a tagged purified product, target denaturation in immobilization, non‐specific surface interactions of library members, and difficulty enriching modest binders. DNA‐programmed photo‐affinity labeling (DPAL) approaches use a photocrosslink to strengthen transient interactions in solution. Interaction Dependent PCR (ID‐PCR) and Interaction Determination using Unpurified Proteins (IDUP) offer the ability to enrich hits from complex mixtures using a primer attached to the target either covalently or noncovalently.

The most common strategy for the enrichment of a library in both mRNA‐display and DELs is by pull‐down approaches, relying on interactions between the displayed library members and the bait protein immobilized on a column, plate, magnetic beads, or other solid support. A series of stringent washing steps is then able to separate potent binders from weak or non‐binders.^[157]^ Despite the relative simplicity and efficiency of this method, it also carries potential drawbacks that include the risk of altering the conformation of the target protein and thereby altering its binding site, inaccessibility of a relevant binding pocket because of a fixed orientation in presentation, difficulty in scaling to higher throughput, and less relevant interactions at a surface rather than in solution, the need for a tagged a purified protein to ensure relevant binders enrich, and the risk that stringency may be too high and no hits are found at all. Furthermore, parallel control experiments are typically required to assess undesired interactions such as false positives that bind to the solid support and washing protocols need to be adjusted if moderate binders are undesirably washed out (Figure 6).

In‐solution methods have been developed for enrichment of hits from DELs to overcome the background noise and washing steps related to immobilized targets, but also to better mimic the natural complex target mixtures, aiming for higher *in vivo* success rates of the obtained hit compounds: DNA‐programmed photo‐affinity labeling (DPAL) uses a cross‐linker on the library to better capture and strengthen transient but relevant interactions, while Interaction Dependent PCR (ID‐PCR)^[158]^ and Interaction Determination using Unpurified Proteins (IDUP)^[159]^ both use a ssDNA fragment attached to the target covalently or non‐covalently (respectively) to only allow binding library members to amplify in PCR. Important to note is that these protocols have yet to be extensively applied, having only been validated with well‐known targets such as avidin and CA‐II using small model libraries, and not yet in a ‘true’ molecular discovery setting.

Using purified proteins but in a solution‐binding setting, the DPAL method establishes a covalent connection between a binding library member and the target upon activation of a photo‐crosslinker attached to a small DNA fragment. The group of Li published three iterations of the DPAL method in selection. In their initial protocol the DNA strand carrying a 5’ photo‐crosslinker is complementary to a primer binding site in the ssDNA encoding the library member, and thus is limited to 3’‐end modified libraries.

Digestion with *exo*I nuclease was used to remove unbound library members, with binding to the target providing some degree of protection from the enzyme, although degradation of binders was still observed.^[160]^ The second approach used splinted ligation to add the cross‐linker in a way that generated a hairpin and thereby allows 5’ end modified libraries, and gel purification to separate out binders.^[161]^ The third approach built on the first, but instead used DNA polymerase extension and gel purification to distinguish binders and simplify hit isolation without degrading the tags of potentially interesting molecules.^[162]^ Notably, gel excision following electromotility shift has been applied in mRNA display to find sequences that enhance the display process itself for antibodies,^[10]^ but to the best of our knowledge not for enrichment of target binding.

In a conceptually similar approach developed by the Krusemark group,^[163]^ a 5’ modified ssDNA primer was used to anneal to the 3’ end of a ssDNA extension of a dsDNA‐tagged library. This can then be photoactivated to cross‐link to the target before protein unfolding and pulldown for stringent washing. Although being limited to 5’‐end modified libraries, it does offer a way to use DPAL selection for dsDNA libraries. These approaches provide the ability to use completely unmodified targets and also the ability to capture moderate binders more efficiently due to the covalent linkage between library member and the target. Furthermore, little non‐specific crosslinking was observed in these DPAL methods, attributed to crosslinking being proportional to ligand affinity towards the target,^[163]^ (a high effective molarity is present for the photo‐crosslinker only in cases of binding). A similar idea has been demonstrated for mRNA‐displayed peptides (using the cDNA display approach) where a photo‐crosslink is formed between a short oligonucleotide on the target protein and a complementary sequence introduced into the puromycin linker (similar to site 4 in section 4.2 above, although not at the extreme 3’ end).^[17]^ This has not yet been used to enrich target binders, but clearly demonstrates the feasibility of using other crosslinker‐based approaches in mRNA display.

Selection against a target directly in a cellular setting has been achieved by mRNA display against a G‐protein coupled receptor, using CHO cells not expressing the target for counter‐selection of non‐specific binders.^[164]^ A further evolution of the photocrosslinking approaches in the preceding paragraphs has been developed by which a DEL can be selected against a protein target in even an intracellular setting.^[165]^ In this, a cyclic cell‐penetrating peptide is added to the library to deliver it into the cell. Once inside the cell, the same photocrosslinking idea of DPAL is used to make the connection of any binding library members to the target more stable before purification of the target protein by affinity tag and amplification of any attached DNA to identify binding library members. Given the use of a peptide sequence to deliver this library into the cell, its implementation in mRNA display seems plausible (provided that the single stranded mRNA tag is stabilized; cDNA‐TRAP or cDNA display would be especially well suited).

In an ID‐PCR approach the binding of a library member to the target protein brings it into close proximity of an oligonucleotide, which acts as a primer for polymerase extension only in the case of target binding.^[158]^ This builds on the photocrosslinker‐based enhancement of binders in DPAL to remove the need for a pull‐down or gel purification step. Instead, only interacting sequences are able to be PCR amplified. This makes it much more amenable to high‐throughput application to multiple targets. In this approach, a target protein is modified with a binding probe (BP), an oligonucleotide which carries a known ligand of the protein of interest, and a capture probe (CP), another oligonucleotide which bears a crosslinking group.^[166]^ Upon engagement of the BP/CP duplex with the target and subsequent irradiation to initiate crosslinking, a displacement probe is used to remove the binding probe through toehold displacement, leaving only the capture probe attached at the site of binding. The capture probe ssDNA sequence is specific for the target and furthermore complementary to a constant primer binding site on all library member DNA‐tags. The original authors envisioned the DNA‐tag to serve as a ‘homing beacon’ which not only increases affinity by avidity, but also enhances target specificity through site‐specific library hybridization. In an mRNA display setting this would require a single‐stranded sequence complementary to the BP oligo, either in the mRNA or cDNA (e. g. sites 2, 3, 4 or 6 in the previous section).

In contrast to ID‐PCR, IDUP does not necessarily generate a covalent connection with the target.^[159]^ It either relies on covalent self‐labeling using the likes of CLIP‐, Halo‐ or SNAP‐tags which are conjugated to a ssDNA sequence complementary to the primer binding site of the library ssDNA sequence; or by using ssDNA‐tagged antibodies which function the same as the tag‐approach, but do not form a covalent connection. This approach has less free choice of binding site for the homing effect due to the placement of the tag, but it does not require an existing ligand to generate the binding probe. It furthermore provides the ability to encode which target was bound, as demonstrated in a cross‐library screening campaign of a DNA‐tagged small molecule library and a DNA‐tagged target library.^[167]^ Notably, these approaches also allow enrichment in a more complex setting, including cell lysates or on membranes.^[159]^

## 6 Future Directions

As outlined above, literature on DEL approaches offers a wealth of techniques that could be used to enhance mRNA display selections. Chemical modifications are already being implemented in mRNA display selections, and there is a growing body of reactions being used for this. Reactions used in building DELs are for the most part likely inherently compatible, being already validated for compatibility with a DNA tag and for reaction in water at high dilution. Although RNA stability is less than that of DNA, in most cases this is unlikely to be limiting. In cases where it is limiting, transfer of the peptide information into a covalently coupled cDNA tag, as in cDNA display or cDNA‐TRAP, will very likely overcome the problem. The most appealing reactions are those that are efficient in conversion, that allow use of a large pool of building blocks, and that generate a drug‐like linkage (in this context taken to mean minimal additional mass, and with minimal additional H‐bond donors and acceptors). Following these parameters, we consider the most promising to be amide coupling reactions^[8]^ (especially when applied on the *N*‐terminus or a shorter‐chain amino acid such as diaminopropionic acid, to minimize flexibility), Pd‐catalyzed cross‐coupling reactions on iodophenylalanine,^[8]^ *N*‐Terminal modifications either with aldehydes^[129]^ (especially reagents that react further such as 2‐pyridinecarboxaldehydes) or following generation of an aldehyde using periodic acid, CuAAc reactions^[8]^ and Larock indole annulation.^[121]^ Use of an increasing diversity of modification reactions requires approaches to track these modifications, and we have outlined six possible loci where such information could be encoded in an mRNA display construct, building on the precedent for additional functionality in the puromycin linker used in cDNA display and for silent encoding of modifications in phage display. Finally, we outline several alternate approaches that could be used to enrich binders beyond the traditional approach of an immobilized protein, which may overcome limitations in some cases.

Adoption of these ideas can lead to the creation of hybrid approaches that we believe will offer access to discovery of heavily modified small peptides that may address some of the issues with peptides in drug development. In these, the peptide component will likely be shorter than a typical mRNA display experiment at present, still serving as a source of functional groups but importantly also serving as a highly adaptable scaffold for presentation of additional moieties not present in peptides.

**Figure 7:**
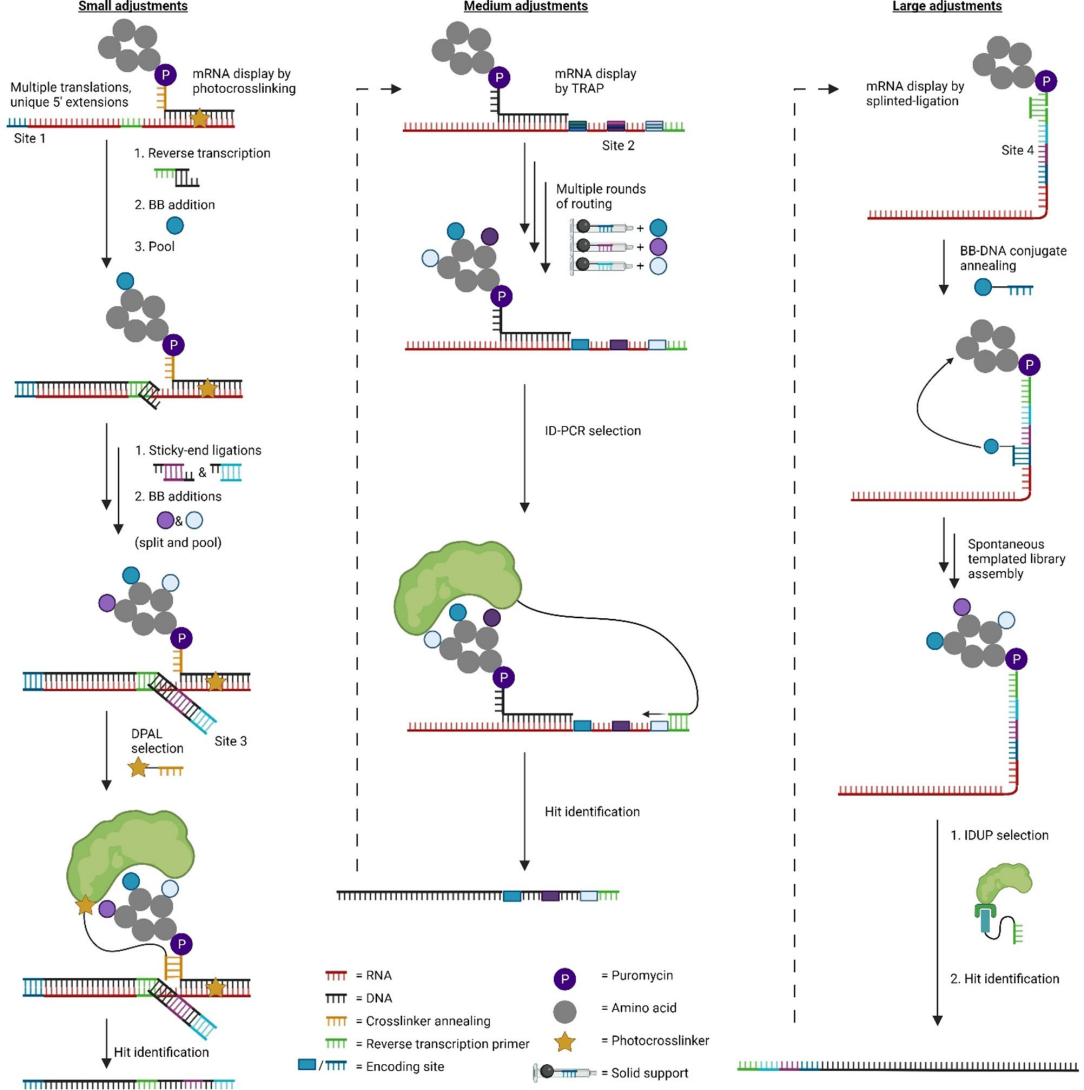
Example potential future convergence of mRNA display and DNA‐encoded libraries in a hybrid approach, with small, medium and large adjustments from current protocols. The use of alternate enrichment approaches is not necessary for these implementations, but represent additional restrictions on library generation and so are shown here to illustrate compatibility. See text for descriptions.

We conclude by outlining three more concrete examples of how we imagine the above ideas could be combined with small, medium and large modifications to the current format (Figure 7).

With only small modifications, a DNA‐recorded approach could immediately be implemented. In a Y‐ligation or photocrosslinked approach to mRNA display, a reverse transcription primer can be added before enrichment of binders. Adding one of a diverse set of oligonucleotides with a conserved 3’ annealing region and a varied 5’ encoding region could record a first reaction (site 3). With an additional short conserved 5’ region this could then be built on by ligation to record a further series of reactions in a split‐and‐pool approach. Where needed to strengthen interactions of weaker hits, DPAL‐type binding enrichment could additionally be achieved in this case with a short oligonucleotide included in the puromycin linker bringing the photocrosslinker and library into the required proximity with the target. This would, however, not be amenable to multiple sequential rounds for molecular evolution.

In a more elaborate method, an extension of the mRNA template at either the 5’ end in a photocrosslinking approach or either the 5’ or 3’ end in a TRAP display approach (sites 1 and 2) could be exploited for encoding by routing. A library diversified with a set of tags at the 3’ end could be captured on complementary oligonucleotides in such an mRNA‐routed approach to encode a series of modification reactions. A further conserved 3’ extension would then be a suitable handle for ID‐PCR or IDUP enrichment of binders, if desired. This could be applied in a multiple sequential round format for molecular evolution as the required tags are regenerated after amplification of binders.

Finally, with more extensive modification of the mRNA display platform, a fully templated approach would be particularly powerful, with both translation and subsequent modification reactions taking place in one pot without additional handling steps beyond adding oligonucleotide‐tagged reagents.^[153]^ This could be achieved using additional information encoded in the puromycin linker (site 4) in a Y‐ or splinted ligation approach. A set of ‘codons’ could each template a set of modification reactions driven by proximity to the peptide, and thus high effective concentration. This would take significantly more work to establish, but once implemented would be easy to carry out (and thus fast and able to be run in parallel for high‐throughput discovery). We believe that using a variant of the splinted ligation approach, with a ligation site relatively close to the puromycin, would potentially also allow for multiple sequential rounds of evolution, with the ‘codons’ for both peptide translation and its subsequent modification being coupled in a single oligonucleotide that together acts as a template for the final molecule. A short, conserved region at the extreme 3’ end (represented in green in the figure) could serve as both the splint binding site for ligation as well as reverse transcription primer binding site. With this, any of the outlined selection approaches could be used, and would give true directed evolution of druglike peptide‐small molecule hybrids.

## Conflict of interest

The authors declare no conflict of interest.

## Biographical Information

*Paddy Melsen is a Master's student at the VU Amsterdam, where he began in 2020 after having graduated from the College of Pharmaceutical Sciences honors Bachelor program at Utrecht University. During the final year of his Bachelor's degree he completed an internship in the group of Prof. Dr. Boons, working on the synthesis of a lipid‐linked N‐glycan analogue as a recognition probe for mammalian oligosaccharyltransferase*.

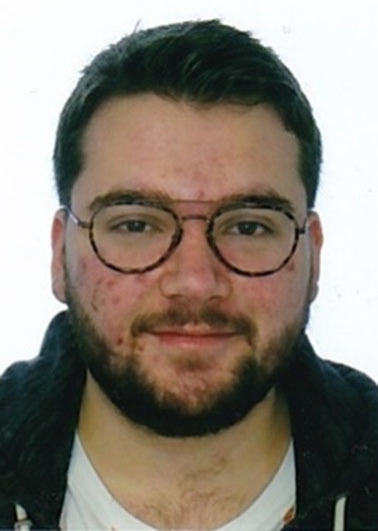

## Biographical Information

*Ryoji Yoshisada is a Ph.D. candidate in the department of Chemistry and Pharmaceutical Sciences (VU Amsterdam). He received his B.Sc. from Kyoto University, with an exchange to Utrecht University in his final year for a research project with Dr. Jongkees. During his M.Sc. studies at Leiden University he was involved in two computational chemistry projects under the supervision of Prof. Fonseca Guerra and Prof. Codée. His current research for his Ph.D. focuses on mRNA display for screening glycopeptide ligands against immunoproteins*.

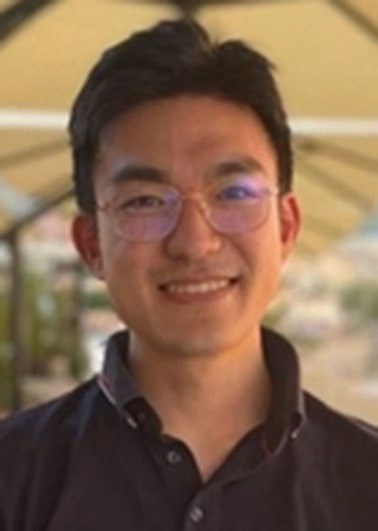

## Biographical Information

*Seino Jongkees is Assistant Professor in the department of Chemistry and Pharmaceutical Sciences at the Amsterdam Institute of Molecular and Life Sciences, VU Amsterdam. He received his B.Sc. and BA from Otago University (New Zealand), then pursued a Ph.D. at the University of British Columbia working with Prof. Withers. Subsequently, he took a JSPS postdoctoral fellowship at the University of Tokyo with Prof. Suga. In 2016 he moved to Utrecht University to establish his own research group before being recruited to VU Amsterdam at the beginning of 2021. Research in the group is focused on the discovery of peptide ligands for carbohydrate‐active enzymes and carbohydrate‐binding proteins as inhibitors and probes, as well as exploring new reactions for diversification in mRNA display*.